# Supplementary material for: Germanium-lead perovskite light-emitting diodes
Source: Nat Commun. 2021 Jul 13;12:4295. doi: 10.1038/s41467-021-24616-5 (PMC8277869; doi:10.1038/s41467-021-24616-5)
Supplement: Supplementary file 1 — Supplementary Information [file 41467_2021_24616_MOESM1_ESM.pdf]

Supplementary Information for:

Germanium-lead perovskite light-emitting diodes

Dexin Yang[1,2#*], Guoling Zhang[1#], Runchen Lai[1], Yao Cheng[2], Yaxiao Lian[1], Min Rao[2], Dexuan Huo[2], Dongchen Lan[3], Baodan Zhao[1,4,5], Dawei Di[1,4,5*]

1. State Key Laboratory of Modern Optical Instrumentation, College of Optical Science and Engineering; International Research Center for Advanced Photonics, Zhejiang University, Hangzhou, 310027, China.
2. College of Materials & Environmental Engineering, Hangzhou Dianzi University, Hangzhou, 310018, China.
3. College of Electrical Engineering, Zhejiang University, Hangzhou, 310027, China
4. Key Laboratory of Excited-State Materials of Zhejiang Province, Zhejiang University, Hangzhou, 310027, China.
5. Cavendish Laboratory, University of Cambridge, JJ Thomson Avenue, Cambridge, CB3 0HE, United Kingdom.

[#] These authors contributed equally.

[*] Corresponding authors: Dawei Di (daweidi@zju.edu.cn); Dexin Yang (dy263@hdu.edu.cn).

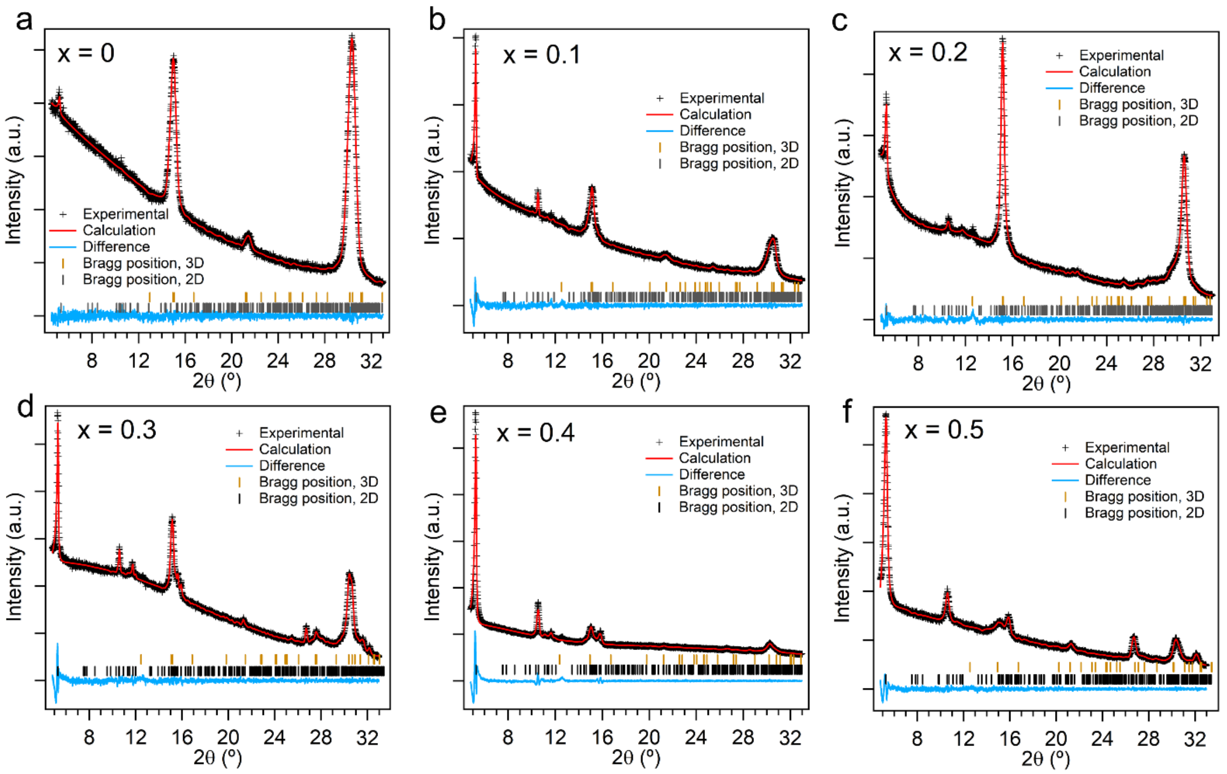

Supplementary Figure 1 | XRD Rietveld refinements of the perovskite films with different Ge inclusion. **a**, with no Ge inclusion. **b**, with 10 mol% Ge. **c**, with 20 mol% Ge. **d**, with 30 mol% Ge. **e**, with 40 mol% Ge. **f**, with 50 mol% Ge. The perovskite samples were spun-coated onto silicon substrates.

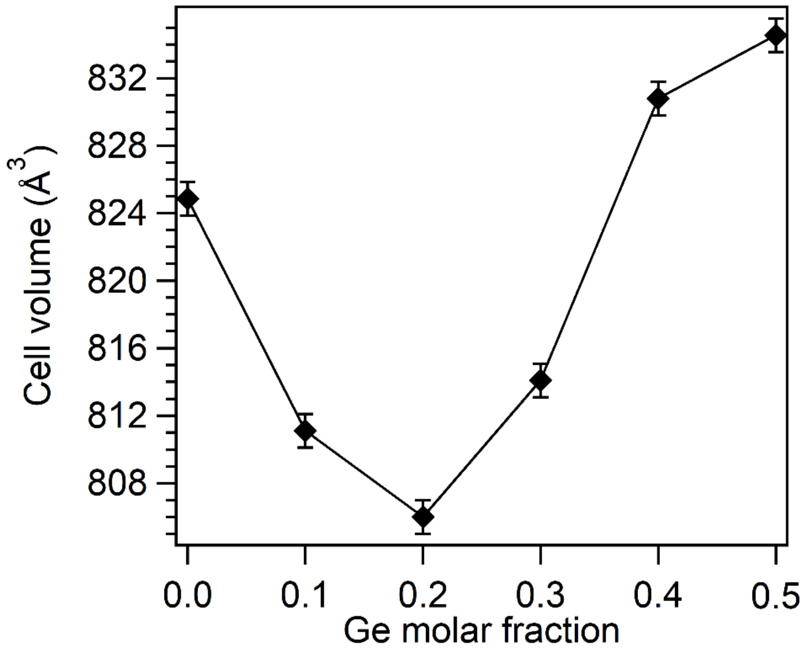

Supplementary Figure 2 | Cell volumes of the $CsPb_{1-x}Ge_xBr_3$ perovskite crystals from the XRD Rietveld refinements. The cell volumes of the perovskite samples decrease when the Ge inclusion increases from 0 mol% to 20 mol%, while the cell volumes increase as the Ge inclusion increases from 20 mol% to 50 mol%. The error bars represent the estimated errors.

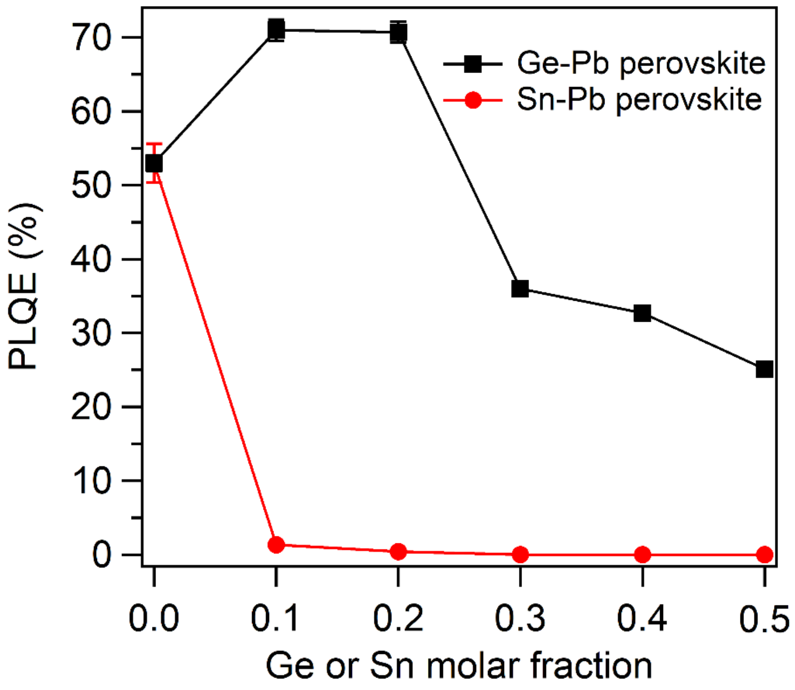

Supplementary Figure 3 | PLQE versus Ge or Sn molar fraction for the perovskite films prepared using the same method. We found that even a small inclusion of Sn decreases the PLQE significantly. The error bars represent the estimated experimental errors.

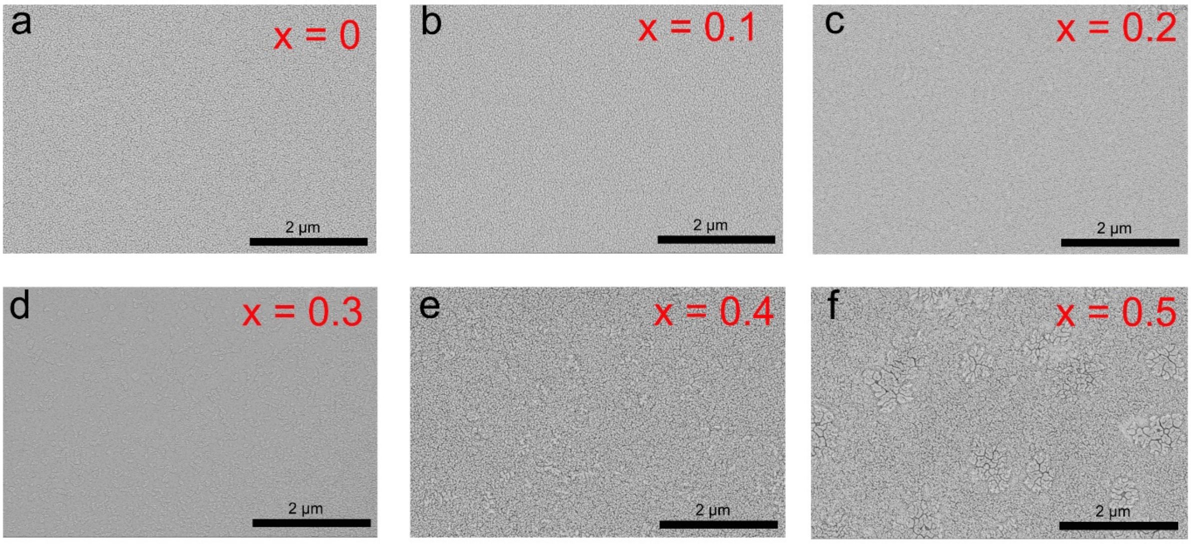

Supplementary Figure 4 | SEM images of the perovskite films with different Ge inclusion. a, with no Ge inclusion. **b**, with 10 mol% Ge. **c**, with 20 mol% Ge. **d**, with 30 mol% Ge. **e**, with 40 mol% Ge. **f**, with 50 mol% Ge. The perovskite samples were deposited on silicon substrates.

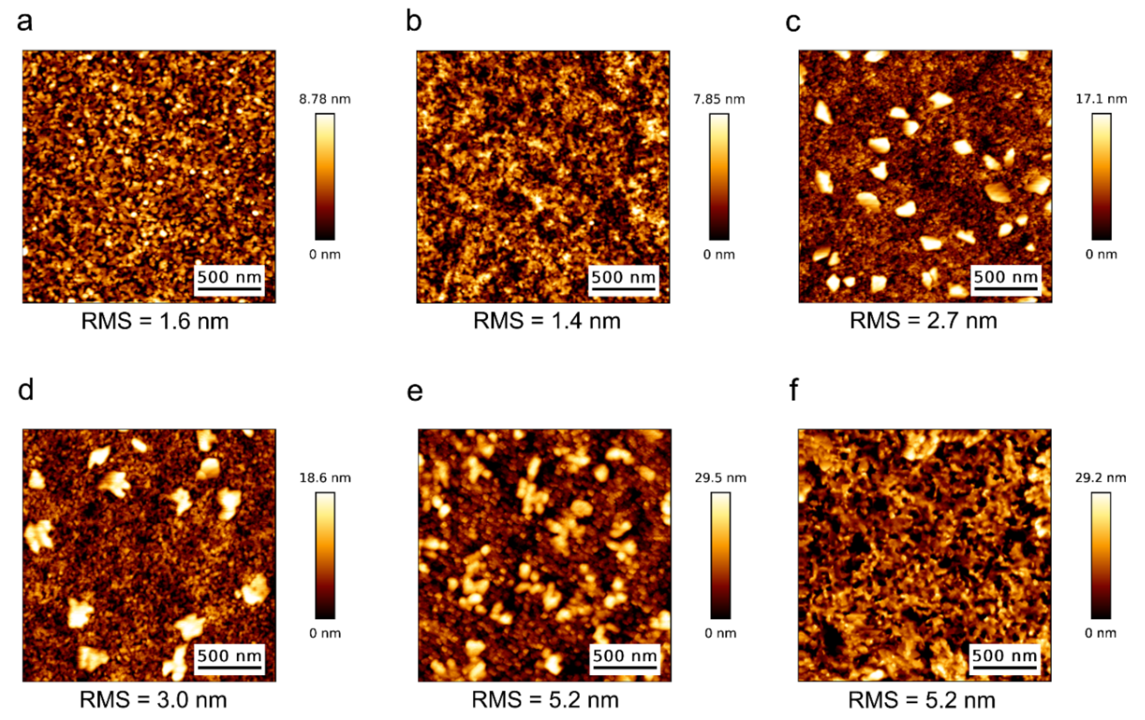

Supplementary Figure 5 | AFM images of the perovskite films with different Ge inclusion. a, with no Ge inclusion. **b**, with 10 mol% Ge. **c**, with 20 mol% Ge. **d**, with 30 mol% Ge. **e**, with 40 mol% Ge. **f**, with 50 mol% Ge. The perovskite samples were deposited on fused silica substrates.

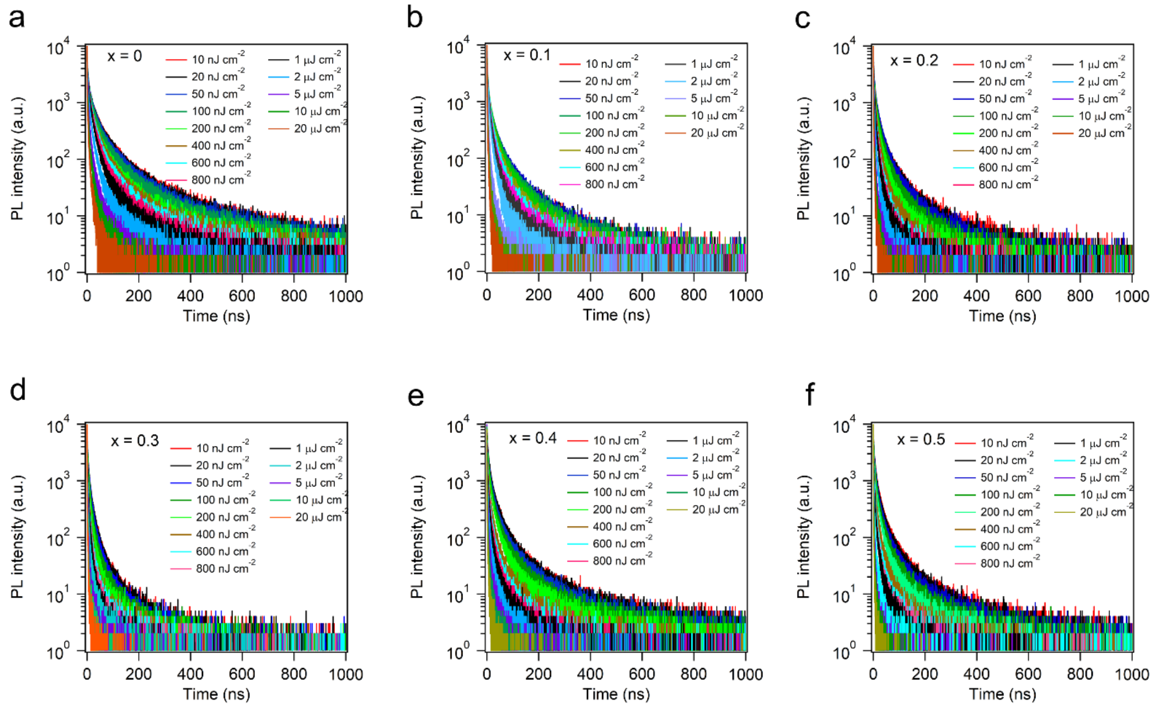

Supplementary Figure 6 | Transient PL decay profiles of the perovskite films with different Ge inclusion under a range of excitation intensities. a, with no Ge inclusion. **b**, with 10 mol% Ge. **c**, with 20 mol% Ge. **d**, with 30 mol% Ge. **e**, with 40 mol% Ge. **f**, with 50 mol% Ge. The perovskite samples were deposited on fused silica substrates.

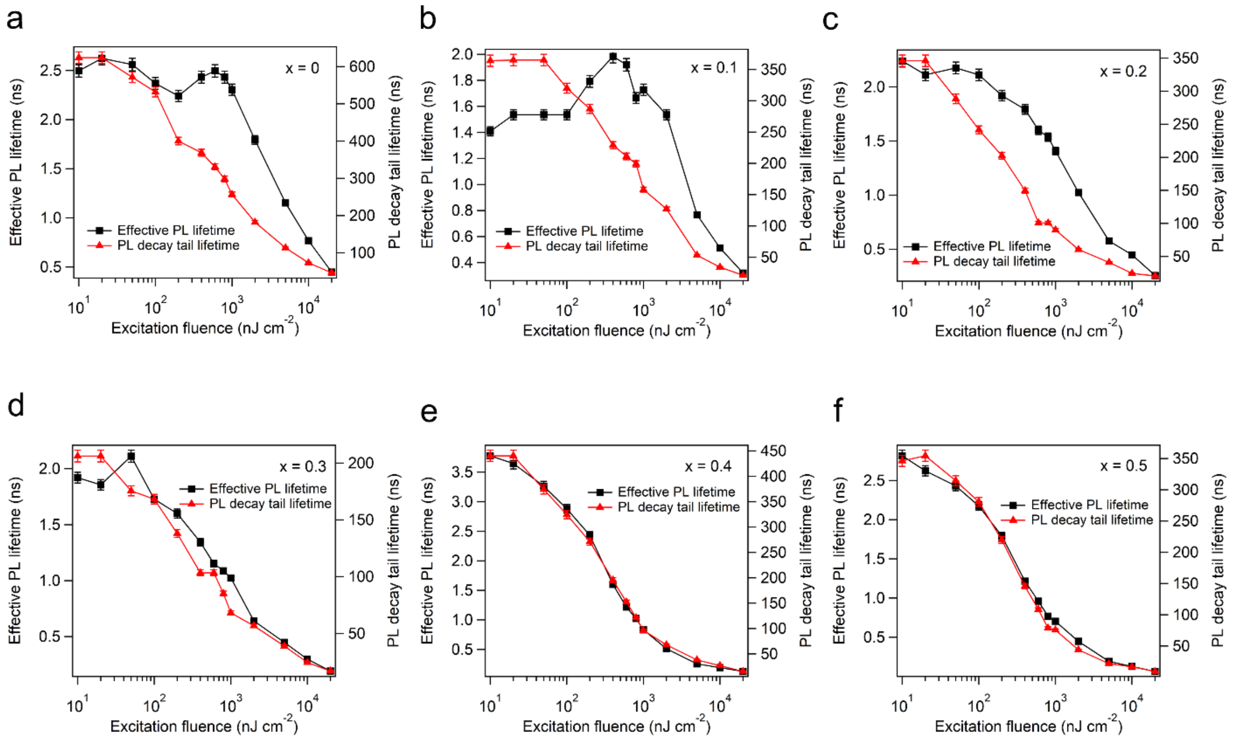

Supplementary Figure 7 | Effective PL lifetimes and PL decay tail lifetimes as functions of excitation intensity for the Ge-Pb perovskite samples. a, with no Ge inclusion. **b**, with 10 mol% Ge. **c**, with 20 mol% Ge. **d**, with 30 mol% Ge. **e**, with 40 mol% Ge. **f**, with 50 mol% Ge. The perovskite samples were deposited on fused silica substrates. The error bars represent the estimated experimental errors.

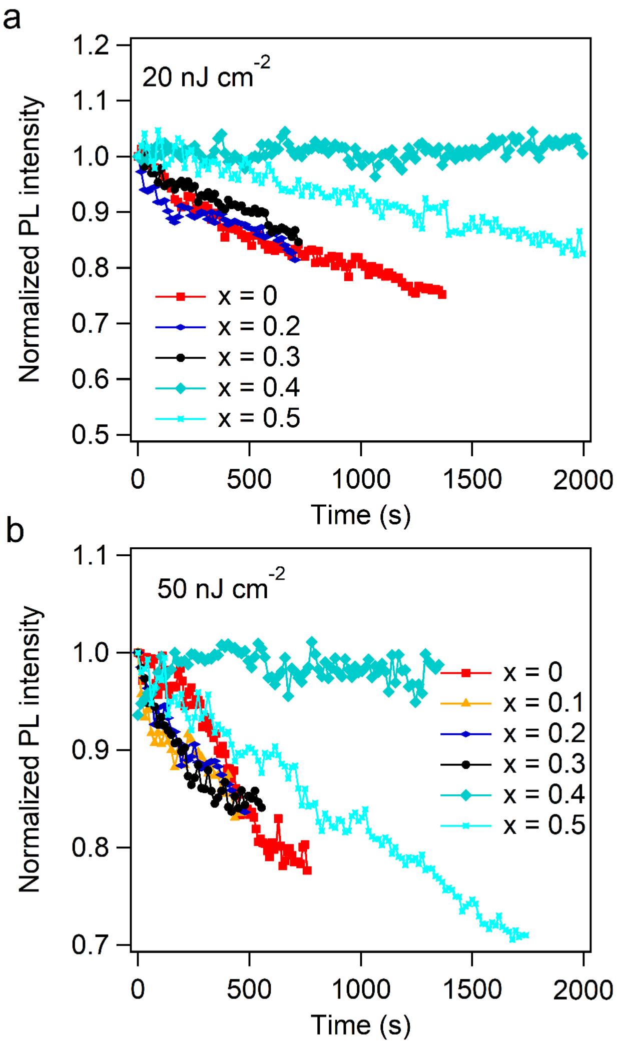

Supplementary Figure 8 | PL stability measurements for the Ge-Pb perovskite films under different excitation intensities. a, 20 nJ cm^{-2}. **b**, 50 nJ cm^{-2}.

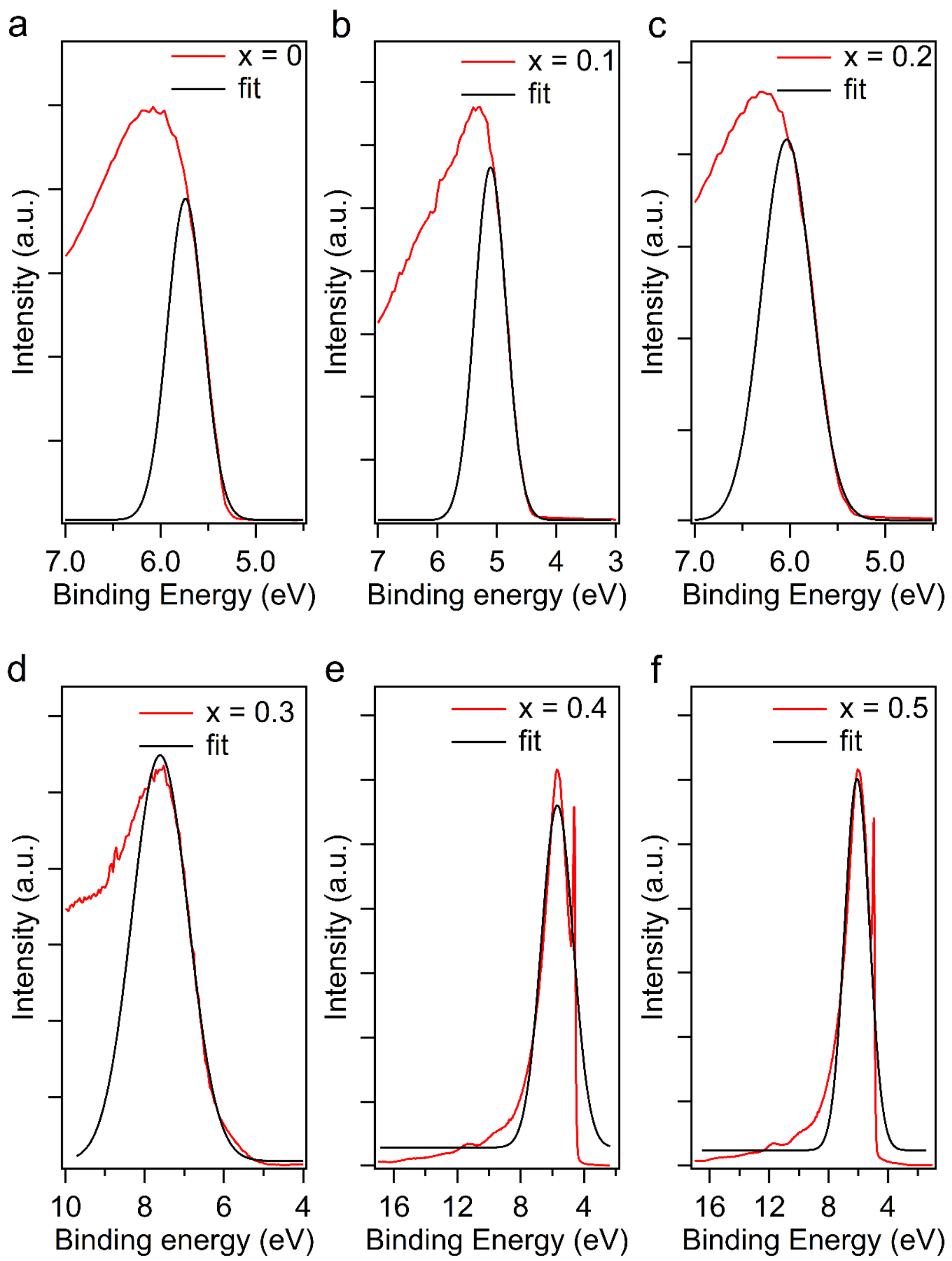

Supplementary Figure 9 | UPS spectra of the perovskite samples. UPS spectra of the perovskite films with different Ge molar fractions. **a,** 0%; **b,** 10%; **c,** 20%; **d,** 30%; **e,** 40%; **f,** 50%. Gaussian fits were used to determine the VBM.

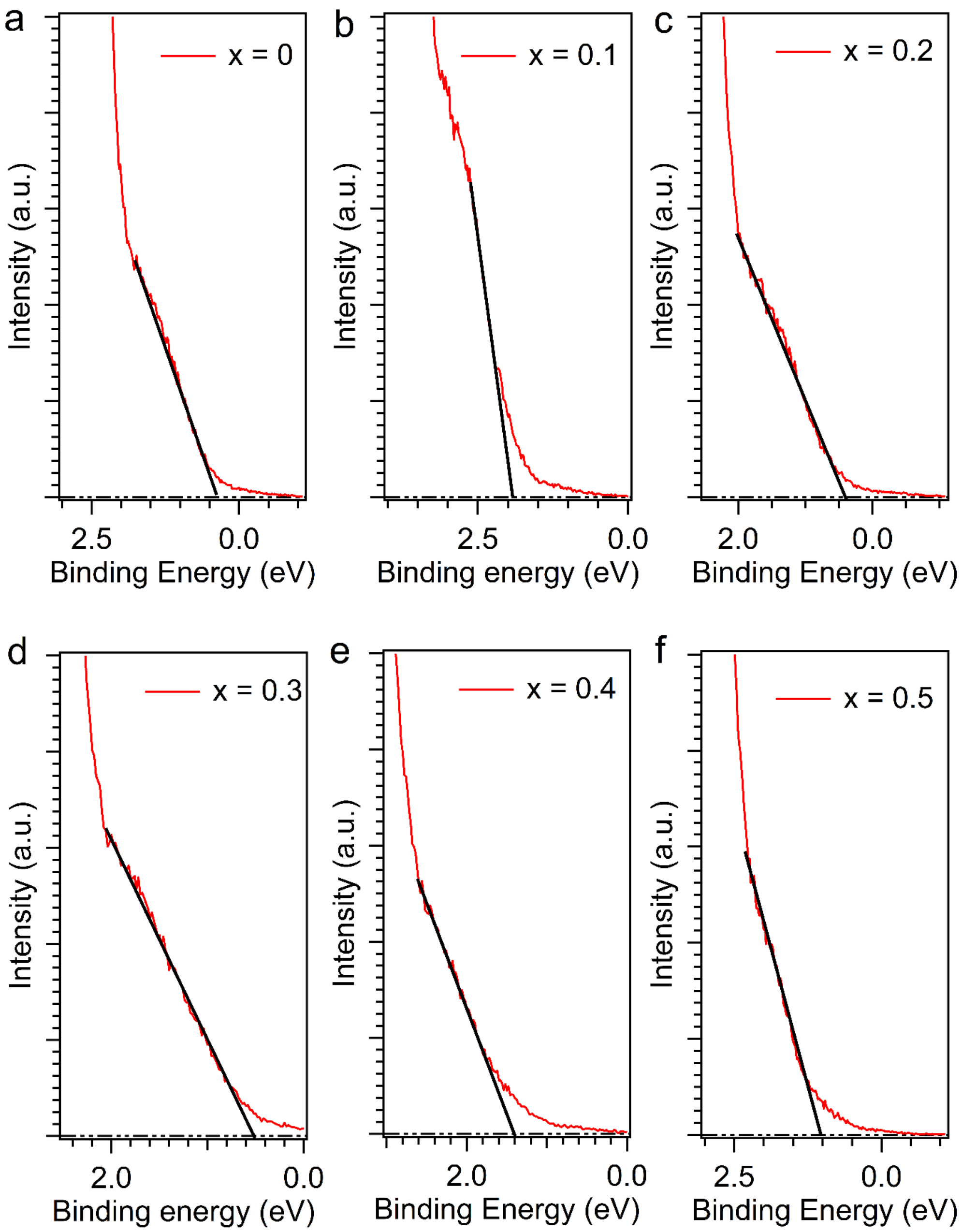

Supplementary Figure 10 | UPS spectra of perovskite samples. The secondary electron spectra of perovskite films with different Ge molar fractions. **a,** 0%; **b,** 10%; **c,** 20%; **d,** 30%; **e,** 40%; **f,** 50%.

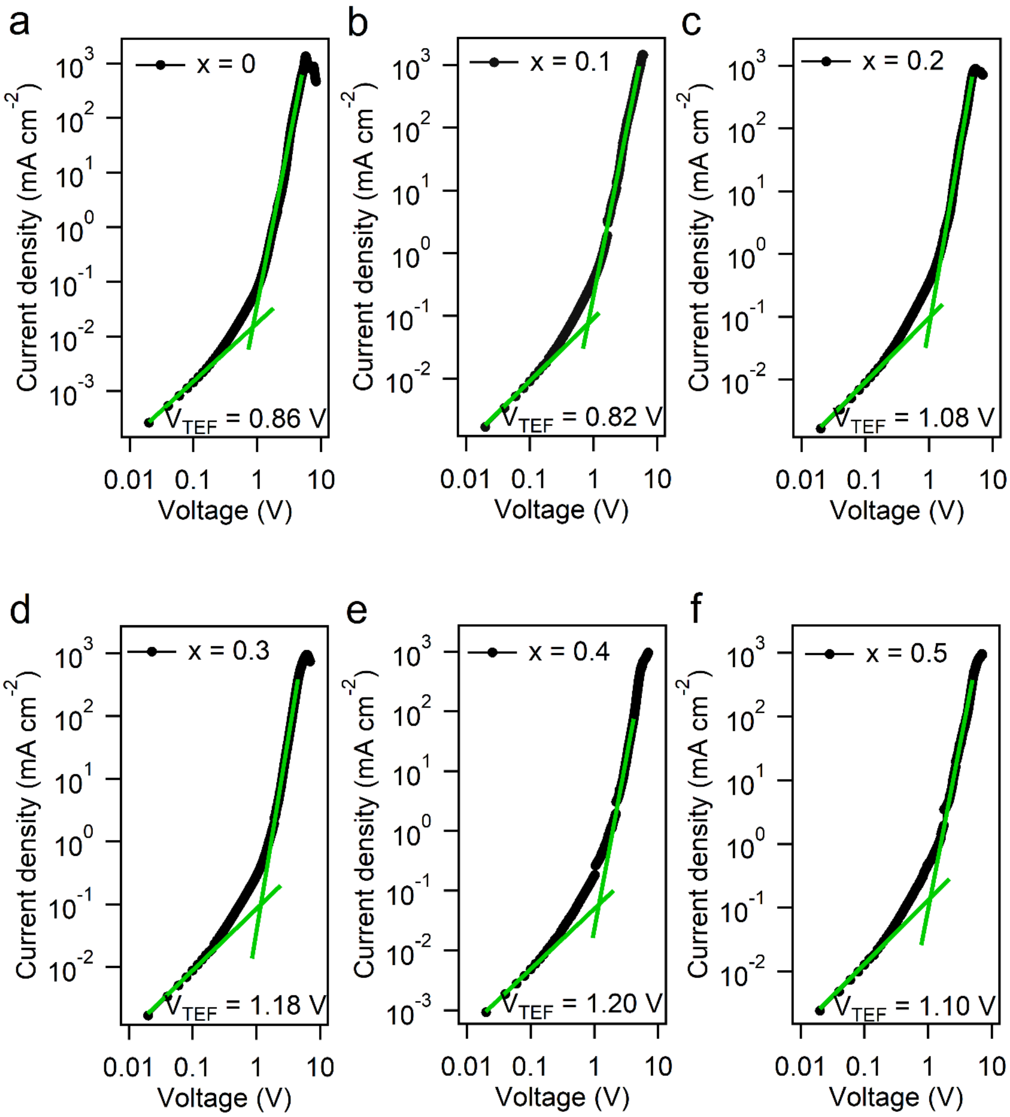

Supplementary Figure 11 | J-V characteristics of hole-only devices based on Ge-Pb perovskite films with the device structures of ITO/PEDOT:PSS/PVK/perovskite/MoO_x/Ag. a, 0%; **b,** 10%; **c,** 20%; **d,** 30%; **e,** 40%; **f,** 50%.

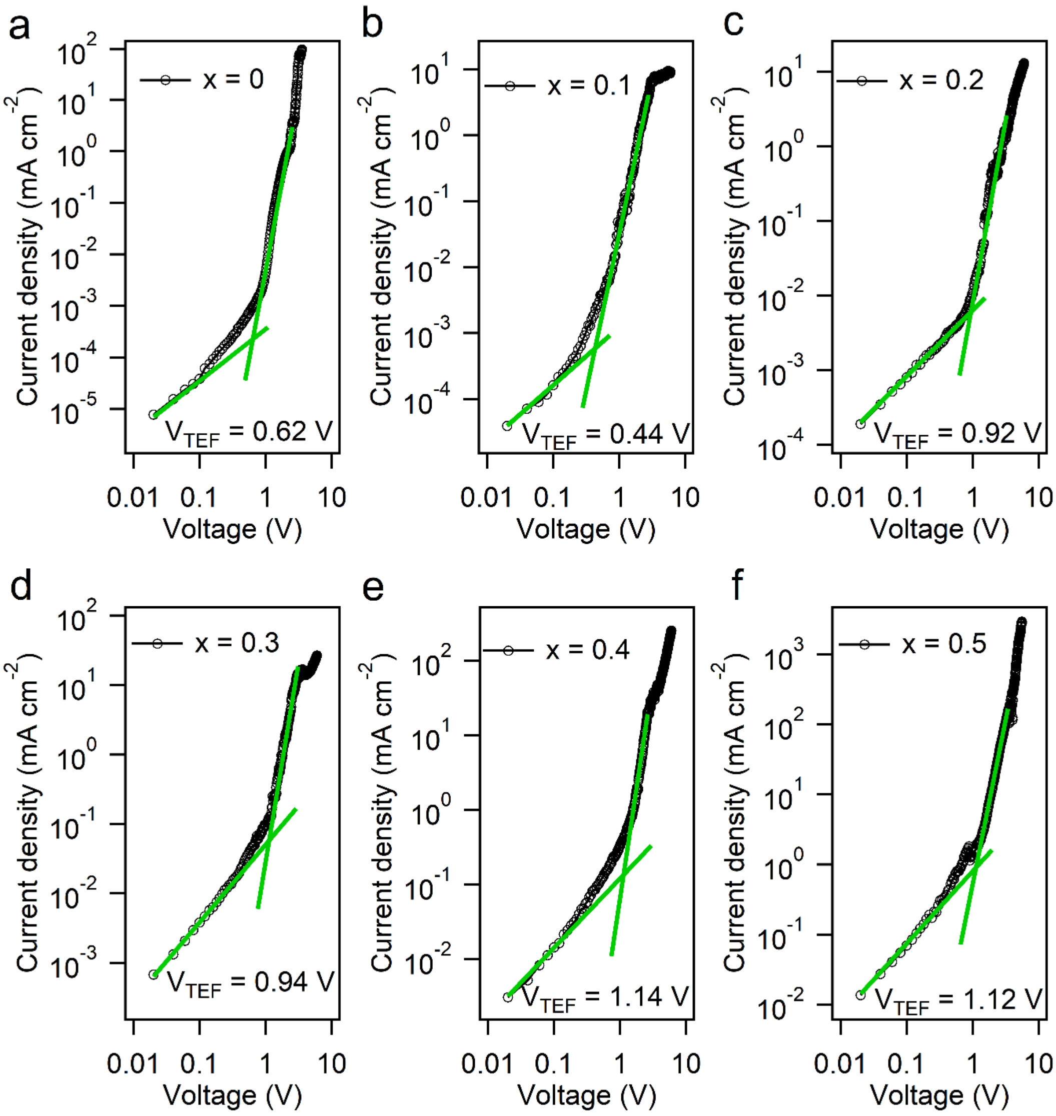

Supplementary Figure 12 | J-V characteristics of electron-only devices based on Ge-Pb perovskite films with the device structures of ITO/TPBi/perovskite/TPBi/LiF/Al. a, 0%; **b,** 10%; **c,** 20%; **d,** 30%; **e,** 40%; **f,** 50%.

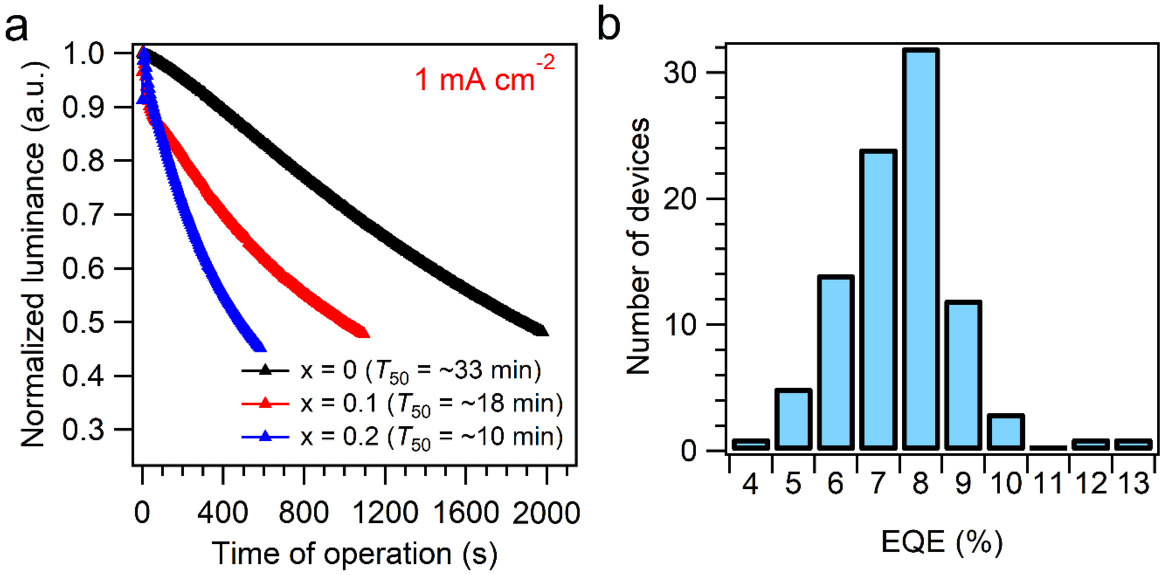

Supplementary Figure 13 | Additional characterization of Ge-Pb PeLEDs. a, Device operational stability tests for the Ge-Pb PeLEDs with 0 mol%, 10 mol%, and 20 mol% Ge inclusion; **b,** peak EQE histogram of Ge-Pb PeLEDs with 10 mol% Ge inclusion.

Supplementary Table 1 | Performance of reduced-toxicity PeLEDs from a selection of recent literature.

Publication date	Perovskite	Device architecture	EL peak (nm)	PLQE of emissive layer	Maximum brightness or radiance	**Peak EQE**	Ref.
2016/07	$CH_3NH_3SnI_3$	ITO/PEDOT:PSS/Pero/F8/Ca/Ag	945	5.3%	3.4 W $sr^{-1}m^{-2}$	**0.72%**	S1
2016/09	$CsSnI_3$	ITO/PEDOT:PSS/Pero/PBD/LiF/Al	950	——	40 W $sr^{-1}m^{-2}$	**3.8%**	S2
2017/10	$CsPb_{0.67}Sn_{0.33}Br_3$ QDs	ITO/PEDOT:PSS/TFB/Pero/TPBi/LiF/Al	517	45%	12500 cd m^{-2}	**4.13%**	S3
2018/12	$(OAm)_2SnBr_4$	ITO/ZnO/PEI/Pero/TCTA/MoO_3/Al	625	68%	350 cd m^{-2}	**0.1%**	S4
2019/02	$(PEAI)_{3.5}(CsI)_5(SnI_2)_{4.5}$	ITO/PVK/Pero/TmPyPB/LiF/Al	920	18%	40 W $sr^{-1}m^{-2}$	**3%**	S5
2020/01	PEA_2SnI_4	ITO/PEDOT:PSS/Pero/TPBi/LiF/Al	629	0.41%	58 cd m^{-2}	**0.16%**	S6
	TEA_2SnI_4		638	1.52%	322 cd m^{-2}	**0.62%**	
2020/03	PEA_2SnI_4	ITO/PEDOT:PSS/Pero/TPBi/LiF/Al	633	1.35%	70 cd m^{-2}	**0.3%**	S7
2020/06	$CsSnBr_3$	ITO/MoO_3/TAPC/TCTA/Pero/TmPyPB/LiF/Al	674	3.76%	43 cd m^{-2}	**0.16%**	S8
2020/07	$(PEA)_2SnI_4$	ITO/PEDOT:PSS/Pero/TPBi/Al	630	10%	355 cd m^{-2}	**0.52%**	S9
2020/10	$(PEA)_2SnI_4$	ITO/PEDOT:PSS/Pero/TPBi/LiF/Al	632	7%	170 cd m^{-2}	**5%**	S10
2021/05	$PEA_2Cs_{n-1}(Ge_{0.1}Pb_{0.9})_nBr_{3n+1}$ ($1 \leq n \leq \infty$)	ITO/PVK/Pero/TPBi/LiF/Al	514	71%	~10000 cd m^{-2}	**13.1%**	This work

Supplementary Table 2 | Work function (WF), Fermi level (E_F), valance band maximum (VBM) and conduction band minimum (CBM) values of perovskite films with different Ge content.

Ge molar fraction	WF (eV)	E_F (eV)	VBM (eV)	CBM (eV)
0	5.34	-5.34	-5.74	-3.38
10%	3.43	-3.43	-5.11	-2.75
20%	5.63	-5.63	-6.03	-3.66
30%	6.37	-6.37	-7.61	-5.24
40%	4.29	-4.29	-5.69	-3.33
50%	5.05	-5.05	-6.09	-3.72

Supplementary references

S1. Lai M., Tay T., Sadhanala A., Dutton S. E., Li G., Friend R. H. *et al.* Tunable Near-Infrared Luminescence in Tin Halide Perovskite Devices. *J. Phys. Chem. Lett.* **7**, 2653-2658 (2016).

S2. Hong W. *et al.* Efficient Low-Temperature Solution-Processed Lead-Free Perovskite Infrared Light-Emitting Diodes. *Adv. Mater.* **28**, 8029-8036 (2016).

S3. Wang H., Wang W., Tang A. *et al.* High-Performance $CsPb_{1-x}Sn_xBr_3$ Perovskite Quantum Dots for Light-Emitting Diodes. *Angew. Chem. Int. Edit.* **56**, 13650-13654 (2017).

S4. Zhang X., Wang C., Zhang Y., Zhang X., Wang S., Lu M. *et al.* Bright Orange Electroluminescence from Lead-Free Two-Dimensional Perovskites. *ACS Energy Lett.* **4**, 242-248 (2018).

S5. Wang Y., Zou R., Chang J., Fu Z., Cao Y., Zhang L. *et al.* Tin-Based Multiple Quantum Well Perovskites for Light-Emitting Diodes with Improved Stability. *J. Phys. Chem. Lett.* **10**, 453-459 (2019).

S6. Wang Z., Wang F., Zhao B., Qu S., Hayat T., Alsaedi A., *et al.* Efficient Two-Dimensional Tin Halide Perovskite Light-Emitting Diodes via a Spacer Cation Substitution Strategy. *J. Phys. Chem. Lett.* **11**, 1120-1127 (2020).

S7. Liang H., Yuan F., Johnston A., Gao C., Choubisa H., Gao Y. *et al.* High Color Purity Lead-Free Perovskite Light-Emitting Diodes via Sn Stabilization. *Adv. Sci.* **7**, 1903213 (2020).

S8. Mu H., Hu F., Wang R., Jia J., Shuang X.. Effects of in-situ annealing on the electroluminescence performance of the Sn-based perovskite light-emitting diodes prepared by thermal evaporation. *J. Lumin.* **226**, 117493 (2020).

S9. Liao Y., Shang Y., Wei Q., Wang H., Ning Z.. Two-dimensional tin perovskite nanoplate for pure red light-emitting diodes. *J. Phys. D. Appl. Phys* **53**, 414005 (2020).

S10. Yuan F., Zheng X., Johnston A., Wang Y., Zhou C., Dong Y. *et al.* Color-pure red light-emitting diodes based on two-dimensional lead-free perovskites. *Sci. Adv.* **6**, eabb0253 (2020).
